# Age-adjusted global glomerulosclerosis predicts renal progression more accurately in patients with IgA nephropathy

**DOI:** 10.1038/s41598-020-63366-0

**Published:** 2020-04-14

**Authors:** Chan-Sung Chung, Ji-Hye Lee, Si-Hyong Jang, Nam-Jun Cho, Wook-Joon Kim, Nam Hun Heo, Hyo-Wook Gil, Eun Young Lee, Jong-Seok Moon, Samel Park

**Affiliations:** 1 Department of Internal Medicine, Soonchunhyang University Cheonan Hospital, Cheonan, Korea; 2 Department of Pathology, College of Medicine, Soonchunhyang University, Cheonan, Korea; 3 Department of Biostatistics, Soonchunhyang University Cheonan Hospital, Cheonan, Korea; 4 Institute of Tissue Regeneration, College of Medicine, Soonchunhyang University, Cheonan, Korea; 5 Soonchunhyang Institute of Medi-bio Science, Soonchunhyang University, Cheonan, Korea

**Keywords:** Medical research, Nephrology

## Abstract

The Oxford classification was developed to predict the outcome of IgA nephropathy (IgAN). Based on the upper reference limit (95^th^ percentile) for the number of globally sclerotic glomeruli (GSG) expected on biopsy according to age, we evaluated whether the prognosis of IgAN was affected by the age-calibrated numbers of GSG independent of the Oxford classification. Patients diagnosed with IgAN on renal biopsy in a single center from January 2011 to December 2018 were analyzed retrospectively. Patients with more GSG number than the upper reference limit expected on biopsy according to age were categorized in a group of GSG abnormal for age. We analyzed in two ways, calculating the median rate of decline in estimated glomerular filtration rate (eGFR) and time-to-event defined as a decline of eGFR level to 40% lower than the baseline. There were 111 patients in the group of GSG abnormal for age. In this group, the rate of eGFR decline was faster by 1.85 (3.68–0.03) ml/min/1.73 m^2^ per year in the fully-adjusted robust regression model. The adjusted hazard ratio for eGFR decline for renal outcome was 29.10 (2.18–388.49). The cumulative incidence of CKD progression was significantly higher, especially for those with T score of 0 in the Oxford classification. We suggest that GSG abnormal for age is an independent risk factor in predicting the renal outcome of IgAN.

## Introduction

During the past several decades, the burden of chronic kidney disease (CKD) has increased worldwide^1^. As a cause of end-stage renal disease (ESRD), chronic glomerulonephritis has declined gradually, however, it still accounts for 8.2% of ESRD^2^. In the United States, IgAN is a major cause of CKD and it has been found in about 10% of patients undergoing a renal biopsy^3^. In Korea, a similar result has been reported. IgAN is the most prevalent primary glomerulonephritis and that it accounts for 28.3% of the glomerular disease confirmed by biopsy^4^. Similar results have been shown in Japan^5^ and in China^6^.

Because of the poor outcome and various clinical courses of the IgAN, many studies have tried to predict outcomes, including CKD progression and ESRD^7,8^. As a histopathologic risk factor, the Oxford classification was reported in 2009^9,10^. The Oxford classification comprises four categories including mesangial hypercellularity (M), endocapillary hypercellularity (E), segmental glomerulosclerosis (S), and interstitial fibrosis/tubular atrophy (T). Subsequent studies have revealed that the presence of crescents in glomerulus can independently predict prognosis. Thus, C score has been added to the Oxford classification^11^.

Chronic histological abnormalities described as nephrosclerosis, including global glomerulosclerosis, interstitial fibrosis and tubular atrophy (IF/TA, i.e., T score by the Oxford classification), and arteriosclerosis, are also seen in patients with CKD and healthy aging adult^12^. Recently, the upper reference limit (95th percentile) for the number of globally sclerotic glomeruli (GSG) expected on biopsy according to age was determined^13^. Based on age-calibrated reference of upper limits for GSG, the prognosis of CKD is best predicted without depending on IF/TA^14,15^. The Oxford classification includes semiquantitative measures of nephrosclerosis defined by T score. However, it is not age-adjusted. Therefore, the objective of this study was to investigate whether the prognosis of IgAN might be affected by age-calibrated numbers of GSG independent of T score of the Oxford classification.

## Materials and methods

### Study population

Patients diagnosed with IgAN on renal biopsy at Soonchunhyang University Cheonan Hospital (Cheonan, Korea) from January 2011 to December 2018 were screened. Adult patients (≥18 years old) who were followed up for more than one year were included. Data of these patients were reviewed and analyzed retrospectively. The study protocol was reviewed and approved by the Institutional Review Board of Soonchunhyang University Cheonan Hospital (Cheonan, Korea). The requirement of informed consent was waived because of its retrospective study design (IRB-No: 2019-08-026-001) This study was conducted in accordance with the principles of the Declaration of Helsinki.

### Biopsy specimen characteristics

The diagnosis of IgAN was made by two expert pathologists (JH Lee and SH Jang, coauthors of the present study) based on light microscopy and immunofluorescence. Detailed histologic features were also described according to the Oxford classification. If the number of GSG for a patient was more than the upper reference limit (95^th^ percentile) expected on biopsy according to age, the patient was assigned to the group with GSG abnormal for age^13^. Because reference limits were unavailable for patients aged >77 years, those patients with age >77 years were regarded as having the same thresholds as those with age of 75–77 years. All patients were assigned to the following two groups: GSG normal for age and GSG abnormal for age.

### Clinical characteristics and covariates

Patients’ clinical and demographical data were collected by reviewing their electronic medical records. Clinical characteristics included age, sex, body mass index (BMI), serum creatinine, estimated glomerular filtration rate (eGFR) calculated using Chronic Kidney Disease-Epidemiology Collaboration equation^16^, 24-hour urine protein, and history of smoking, diabetes (defined as having hemoglobin A1c level of 6.5% or higher, use of glucose lowering agents, or self-report), and hypertension (defined as using antihypertensive medications or self-report). These laboratory data were collected at the time of renal biopsy. It was considered immunosuppressive therapy when the patient received steroids more than 0.5 mg/kg/day and/or tacrolimus/cyclosporine/cyclophosphamide for more than four weeks.

### Outcome

We analyzed data in two ways: (1) computing the median rate of decline in eGFR, and (2) calculating the time to event. Based on an inference of linear decrease in eGFR, the rate of eGFR decline was calculated using two points between baseline and the last eGFR follow-up. The renal outcome for time-to-event analysis was defined as an eGFR level 40% lower than the baseline eGFR^17^. To overcome the shortcoming of eGFR which could be affected by a small perturbation of creatinine, we defined the development of renal outcome when the reduction was confirmed by repeated measurements at least one month apart.

### Statistical analyses

Clinical characteristics, biopsy findings, and outcomes of patients with GSG abnormal for age were compared to those with GSG normal for age. Categorical data are presented as count (percentage). Continuous data are expressed as mean ± SD or median (interquartile ranges), as appropriate. Groups were compared using Student’s t-test for normally distributed continuous variables, Mann-Whitney test for non-normally distributed continuous variables, and Pearson’s Chi-squared test or Fisher’s exact test for categorical variables.

The rate of eGFR decline was determined using robust regression models. The hazard ratios for renal outcome were calculated using multivariable Cox proportional models. When regression analysis and Cox proportional analysis were performed, the T and C scores were considered binary variables because only three patients and one patient had T2 and C2, respectively. The model was built and calculated as our previous research^18^.

Kaplan-Meier curves were drawn and a log-rank test was performed in a nonparametric way to compare the survival distribution. All statistical analyses were performed using SPSS 25.0 for Windows (SPSS, Inc., Chicago, IL, USA) and R version 3.4.3 (The R Foundation for Statistical Computing, Vienna, Austria).

## Results

From January 2011 to December 2018, 805 cases of renal biopsy were performed at Soonchunhyang University Cheonan Hospital (Cheonan, Korea). A total of 331 patients were diagnosed with IgAN. Among them, 23 patients were excluded because of their age (less than 18 years), and 91 patients were excluded because of their insufficient follow-up days. Finally, a total of 217 adult patients with biopsy-proven IgAN were enrolled for this study.

The group with GSG abnormal for age had 111 patients. Baseline characteristics of subjects in each group are shown in Table 1. There was no significant difference in the total number of glomeruli on biopsy samples (22 [13–35] vs. 23 [15–33]) or the median follow-up duration (3.71 [2.08–5.89] vs. 3.56 [2.25–5.57]) between the two groups. The group with GSG abnormal for age showed higher serum creatinine levels, more proteinuria, more rapid rates of decline in eGFR, more prevalent history of hypertension, and lower baseline eGFR. Among the Oxford classification, M, S, T, and C scores were higher in the group with GSG abnormal for age. The group with GSG normal for age received more immunosuppressive therapy compared to those with GSG abnormal for age, however, there was no statistical significance (20 [18.9%] vs. 15 [13.5%], *p* = 0.284).

**Table 1:** Baseline clinical characteristics between the group with GSG normal for age and the group with GSG abnormal for age.

	GSG normal for age (n = 106)	GSG abnormal for age (n = 111)	P value
Age, year	40.5 (24.5–51.0)	40.0 (33.0–50.0)	0.434
BMI, kg/m^2^	24.4 ± 4.3	24.0 ± 3.4	0.484
Total glomeruli, count	22 (13–35)	23 (15–33)	0.900
GSG, count	1 (0–1)	5 (3–10)	<0.001
Creatinine, mg/dL	0.8 (0.6–1.0)	1.0 (0.8–1.3)	<0.001
eGFR, ml/min/1.73 m^2^	108.2 (88.8–124.3)	80.7 (63.6–108.0)	<0.001
24 hr urine protein, g/day	0.491 (0.262–1.463)	0.763 (0.425–1.681)	0.012
Rates of eGFR decline, ml/min/1.73 m^2^ per year	−2.61 (−7.56–−0.53)	−4.37 (−8.45–−1.67)	0.024
Male, n (%)	59 (55.7%)	67 (60.4%)	0.483
The Oxford classification			
M1, n (%)	37 (34.9%)	76 (68.5%)	<0.001
E1, n (%)	48 (45.3%)	60 (54.1%)	0.196
S1, n (%)	76 (71.7%)	102 (91.9%)	<0.001
T1, n (%)	7 (6.6%)	42 (37.8%)	<0.001
T2, n (%)	1 (0.9%)	2 (1.8%)	
C1, n (%)	24 (22.6%)	42 (37.8%)	0.029
C2, n (%)	0 (0.0%)	1 (0.9%)	
Current smoker, n (%)	16 (15.1%)	19 (17.1%)	0.685
Diabetes, n (%)	6 (5.7%)	5 (4.5%)	0.698
Hypertension, n (%)	25 (23.6%)	46 (41.4)	0.005
Median follow-up duration, year	3.71 (2.08–5.89)	3.56 (2.25–5.57)	0.996
Immunosuppressive therapy, n (%)	20 (18.9%)	15 (13.5%)	0.284

Abbreviations: GSG, globally sclerotic glomeruli; BMI, body mass index; eGFR, estimated glomerular filtration rate; M, mesangial hypercellularity; E, endocapillary hypercellularity; S, segmental glomerulosclerosis; T, tubular atrophy/interstitial fibrosis; C, crescents.

Rates of eGFR decline were more rapid in the group with GSG abnormal for age than those with GSG normal for age (Table 2). In an unadjusted model and in adjusted models with or without the Oxford classification, the statistical significance remained. As a result, eGFR in the group with GSG abnormal for age decreased faster by 1.85 ml/min per 1.73 m^2^ per year than the group with GSG normal for age in the fully adjusted model (Model 3 in Table 2).

**Table 2:** Estimation of rates of decline in estimated glomerular filtration rate using robust regression model for the group with GSG abnormal for age.

	Model 1^a^	Model 2^b^	Model 3^c^
**GSG**			
Normal for age	Reference	Reference	Reference
Abnormal for age	−1.56 (−3.11–0.01)^d^	−2.41 (−4.04–−0.79)^e^	−1.85 (−3.68–−0.03)^d^

Note: The magnitude in rates of eGFR decline of the group with GSG abnormal for age was expressed as changes of ml/min/1.73 m^2^ per year compared to the group with GSG normal for age.

^a^Model 1 was not adjusted for other variables.

^b^Model 2 was adjusted for sex, smoking, hypertension, diabetes, age, body mass index, baseline eGFR, and baseline 24 hr urine protein.

^c^Model 3 was adjusted for Model 2 variables plus the Oxford classification, e.g. M, E, S, T (T0 or T1–2), and C (C0 or C1-2), and immunosuppressive therapy.

^d^P < 0.05; ^e^ P < 0.010.

Abbreviation: GSG, globally sclerotic glomeruli; eGFR, estimated glomerular filtration rate.

**Figure 1:**
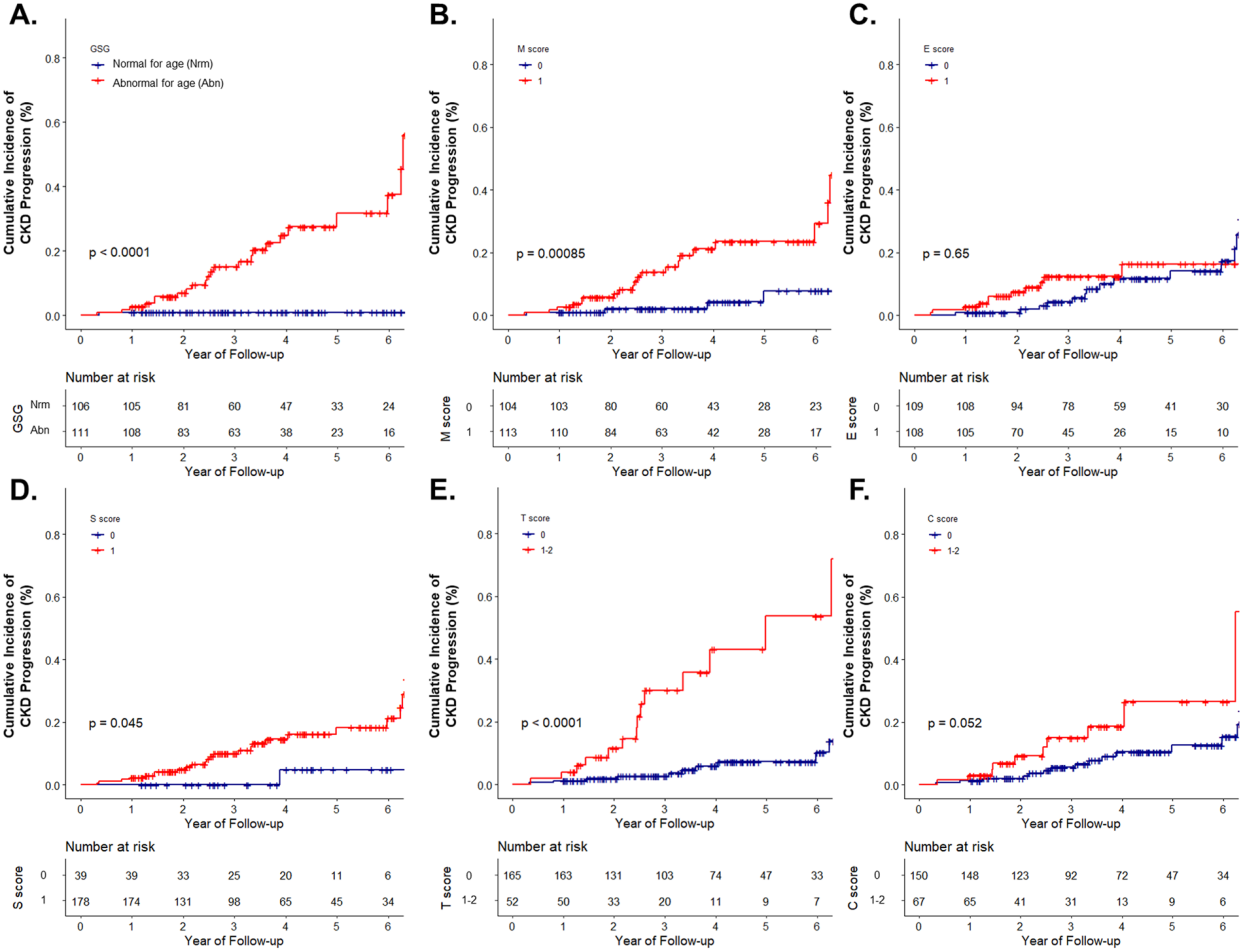
Cumulative incidence of CKD progression defined as a decrease in eGFR by 40% from the baseline, stratified with (**A**) GSG abnormal for age; (**B**) Mesangial proliferation; (**C**) Endocapillary hypercellularity; (**D**) Segmental glomerulosclerosis; (**E**) Tubular atrophy/interstitial fibrosis; and (**F**) Crescents of the Oxford classification. Blue line, score 0; red line, score 1 (red line, score 1–2, when in tubular atrophy/interstitial fibrosis and crescents). Abbreviation: CKD, chronic kidney disease; eGFR, estimated glomerular filtration rate; GSG, globally sclerotic glomeruli.

Figure 1 shows the cumulative incidence of CKD progression (defined as a decline of eGFR level 40% lower than the baseline) according to GSG abnormal for age and MEST-C score of the Oxford classification. Patients in group with GSG abnormal for age showed a higher risk of CKD progression than those in the group with GSG normal for age (P < 0.001, Fig. 1A). As reported previously, M, S, and T score provided information on the prognosis (Fig. 1B,D, and E, respectively). However, E or C score (Fig. 1C and F) did not. The association of GSG abnormal for age with the risk of CKD progression was confirmed in both unadjusted and adjusted Cox regression models (Table 3).

**Table 3:** Association of GSG abnormal for age with the risk of progression of chronic kidney disease.

	Model 1^a^	Model 2^b^	Model 3^c^
**GSG**			
Normal for age	Reference	Reference	Reference
Abnormal for age	30.02 (4.05–222.50)^f^	41.18 (4.44–381.92)^e^	29.10 (2.18–388.49)^d^

Note: Hazard ratios were calculated using the Cox proportional model.

^a^Model 1 was not adjusted for other variables.

^b^Model 2 was adjusted for sex, smoking, hypertension, diabetes, age, body mass index, baseline eGFR, and baseline 24 hr urine protein.

^c^Model 3 was adjusted for Model 2 variables plus the Oxford classification, e.g. M, E, S, T (T0 or T1-2), and C (C0 or C1-2), and immunosuppressive therapy.

^d^P < 0.05; ^e^P < 0.010; ^f^P < 0.001.

Abbreviation: GSG, globally sclerotic glomeruli; eGFR, estimated glomerular filtration rate.

**Figure 2:**
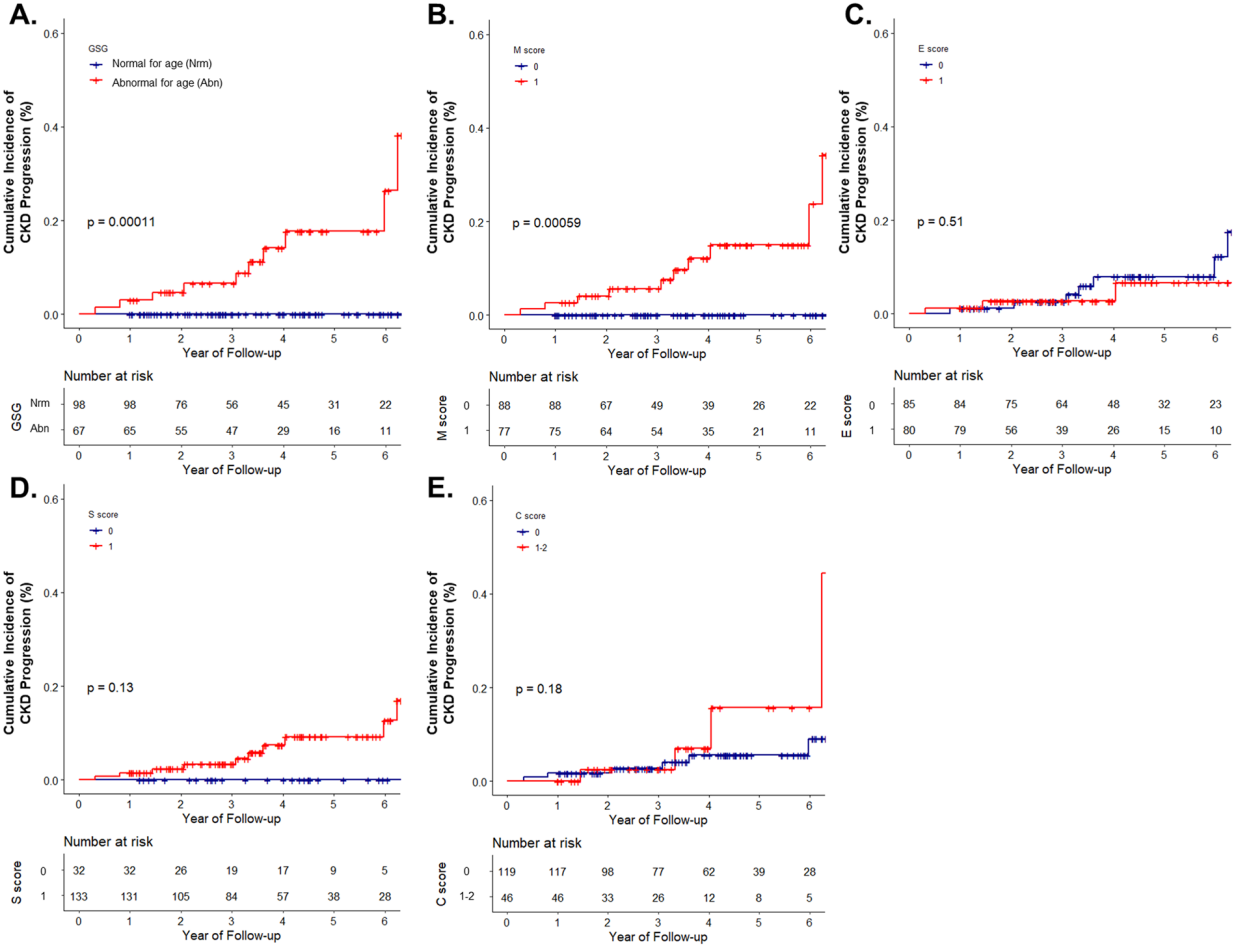
Cumulative incidence of CKD progression in patients with T0. CKD progression was defined as a decrease in eGFR by 40% from the baseline, stratified with (**A**) GSG abnormal for age; (**B**) Mesangial proliferation; (**C**) Endocapillary hypercellularity; (**D**) Segmental glomerulosclerosis; and (**E**) Crescents of the Oxford classification. Blue line, score 0; red line, score 1 (red line, score 1–2, when in crescents). Abbreviation: CKD, chronic kidney disease; eGFR, estimated glomerular filtration rate; GSG, globally sclerotic glomeruli.

**Figure 3:**
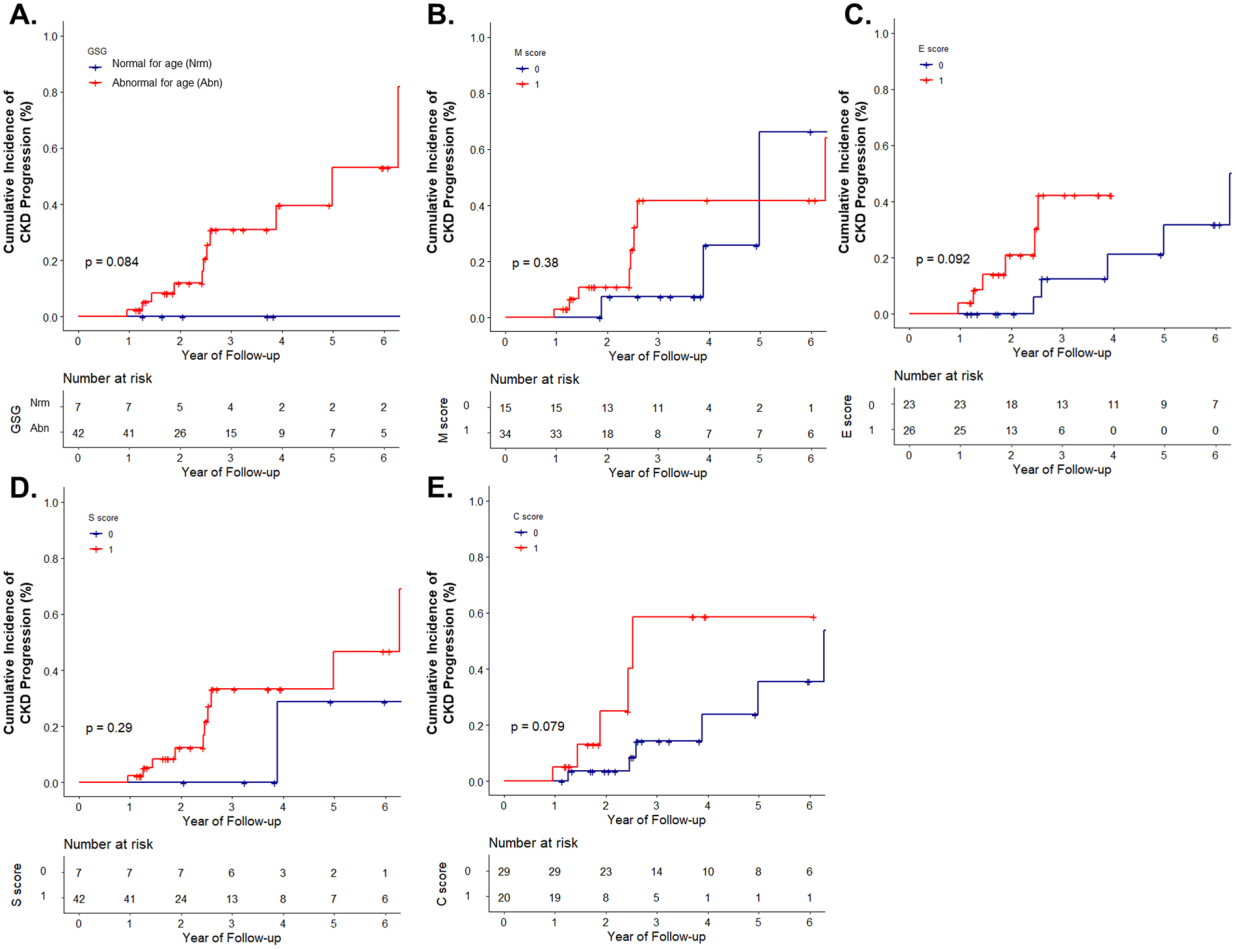
Cumulative incidence of CKD progression in patients with T1. CKD progression was defined as a decrease in eGFR by 40% from the baseline, stratified with (**A**) GSG abnormal for age; (**B**) Mesangial proliferation; (**C**) Endocapillary hypercellularity; (**D**) Segmental glomerulosclerosis; and (**E**) Crescents of the Oxford classification. Blue line, score 0; red line, score 1. Abbreviation: CKD, chronic kidney disease; eGFR, estimated glomerular filtration rate; GSG, globally sclerotic glomeruli.

Patients in the group with GSG abnormal for age had higher T score (P < 0.001, Table 1). Therefore, we analyzed the cumulative incidence of CKD progression according to T score (T0 and T1, presented in Figs. 2 and 3, respectively). The analysis according to T2 was not performed due to the low number of patients with T2 score (n = 3). In addition, all of patients with T2 reached the renal outcome (defined aforementioned) within 4 years (Fig. 1E). In patients with T0, the group with GSG abnormal for age showed a significant cumulative incidence of renal progression compared to the group with GSG normal for age (Fig. 2A). Of the Oxford classification, only the patients with M score of 1 showed a similar result (Fig. 2B). In contrast, in patients with T1, neither the group with GSG abnormal for age nor the Oxford classification showed statistical significance in predicting the prognosis (Fig. 3).

## Discussion

In the present study, we showed that the number of GSG adjusted for age could give information on the prognosis in addition to the Oxford classification. Especially, for patients with T score of 0, the significant progression of IgAN was predicted more precisely. Our results suggested that the number of GSG adjusted for age, as well as the Oxford classification, could be important risk factors in IgAN.

The degree of IF/TA was markedly more severe in patients with GSG abnormal for age compared to those with GSG normal for age (Table 1). In the adjusted robust regression analysis and Cox proportional hazard model, GSG abnormal for age was associated with a more rapid decline in eGFR and it might be a risk factor for renal progression (Tables 2 and 3). In patients with T score of 0, GSG abnormal for age had independent significance in predicting renal outcome (defined as a decrease in eGFR by 40% from the baseline) irrespective of the Oxford classification (Fig. 2). However, this result was not observed in patients with T score of 1 (only showing a tendency, Fig. 3).

In the mechanistic basis of the pathogenesis, close relationships between global glomerulosclerosis and IF/TA have been demonstrated in several studies^10,19,20^. Nephrosclerosis is an irreversible ischemic change shown in renal biopsy. Characteristic features of nephrosclerosis include global glomerulosclerosis, IF/TA, and arteriosclerosis^21^. Nephrosclerosis has been observed to be increasing with age in healthy adults without kidney disease^12^. However, it reflects renal prognosis in CKD patients^22,23^. Fibro-intimal hyperplasia in small arteries with aging leads to global glomerulosclerosis and the corresponding tubules become atrophic with surrounding interstitial fibrosis^20^. In addition, a recent study has suggested that reduction in podocyte density throughout life in humans can cause hypertrophic podocyte stress in some glomeruli, eventually resulting in glomerular tuft collapse and periglomerular fibrosis^19^. This mechanism may be a significant cause of ESRD in the aging kidney and all progressive glomerular diseases can be considered superimposed accelerators of this underlying process.

There has long been an interest in distinguishing age-related glomerulosclerosis from disease-related glomerulosclerosis^24^. However, as a biopsy finding, age-related glomerulosclerosis may be difficult to distinguish from chronic changes due to specific kidney diseases^21^. At the time of developing the Oxford classification, the working group did not test the predictive value of GSG because it was highly correlated to IF/TA. Besides, at that time, it was not possible to separate global glomerulosclerosis associated with age from those associated with IgAN. Among the histology of IgAN, IF/TA is the most important indicator for renal function decline and associated with global glomerulosclerosis. In addition, quantification of the number of GSG is difficult because of the inevitable error caused by a paucity of glomeruli or by subcapsular sampling. In this regard, the Oxford classification includes IF/TA as the T score rather than the number of GSG^9,10^.

However, the upper reference limit (95^th^ percentile) for the number of GSG expected on biopsy according to age was determined in a study of histology from living kidney donors^13^. Numbers of GSG that exceed these thresholds imply pathologic changes that explain not only aging but also underlying glomerular disease. Using these age-calibrated reference limits for global glomerulosclerosis, the risk for renal disease progression is best identified independent of IF/TA in patients with nephrotic syndrome and those with many other kidney diseases^14,15^. Given these contexts, there is a growing interest in age-adjusted GSG numbers that could give additional information for prognosis beyond the Oxford classification, T score^25^.

In our study, patients with GSG abnormal for age had more severe proteinuria. This finding might be attributed to severe disease documented with higher scores in the Oxford classification (Table 1). Given these previous studies, there are emerging controversies regarding the relationship between global glomerulosclerosis and proteinuria. In a previous study on focal segmental glomerulosclerosis, membranous nephropathy, and minimal change disease, patients with GSG abnormal for age were found to have less proteinuria^15^. The association between global glomerulosclerosis and lower proteinuria might be caused by a decline in glomeruli known to filter protein^26^. In contrast, another study has shown that patients with a higher percentage of global glomerulosclerosis have more proteinuria^14^. IgAN with proteinuria of the nephrotic range had more global glomerulosclerosis^27^, showing concordance with our findings that patients with GSG abnormal for age had more proteinuria. Because global glomerulosclerosis associated with aging does not affect proteinuria^12^, these findings suggested that the relationship between proteinuria and global glomerulosclerosis might be associated with the pathophysiology of the disease itself. A recent study has shown that specific type of global glomerulosclerosis, solidification rather than obsolescence, is associated with IgAN-related clinical parameters^27^.

In our cohort comprised of 217 patients, 35 (16.1%) patients received immunosuppressive therapy. There was no statistical difference between the two groups whether immunosuppressive therapy was performed (Table 1). The amount of proteinuria in patients who received immunosuppressive was higher compared to those who did not (1.831 [0.457–4.036] vs. 0.592 [0.324–1.282], respectively). In a fully-adjusted Cox proportional model (Table 3), immunosuppressive therapy was not a factor affecting prognosis (HR: 0.40 [0.13–1.22]). It was attributed to that because only 28% of patients with 1 g/day or more of proteinuria were treated. The reason for the small number of patients who received immunosuppressive therapy was that some patients were included in our study before the publication of guidelines for glomerulonephritis by the Kidney Disease: Improving Global Outcome^28^. Additionally, our institution was concerned about the risk more than the benefit from immunosuppressive therapy. This concern remains after two significant randomized control studies recently published^29,30^.

Our study had several limitations. First, this was a retrospective study performed only in a single center in Korea. Second, all subjects in the present study were Asian. The age-based reference range for GSG was derived from healthy living donors and most of them were White. Whether these reference ranges should differ by race is unclear^13^. Third, creatinine based eGFR was used as an indicator of renal function. Because almost all patients in this study showed nearly normal eGFR (CKD stage 1 or 2), a small perturbation in creatinine level could cause severe changes in eGFR. To overcome this shortcoming, the renal outcome was defined as an eGFR level 40% lower than baseline eGFR lasting more than a month.

In conclusion, GSG abnormal for age might be an independent risk factor that should be evaluated to accurately predict renal outcome of IgAN, especially when T score of the Oxford classification is zero.
